# Knowledge on Applications of 3D Design and Printing in Dentistry Among Dental Practitioners in Saudi Arabia: A Questionnaire-Based Survey

**DOI:** 10.7759/cureus.28379

**Published:** 2022-08-25

**Authors:** Mahesh Suganna, Hina Kausher, Abbasi Begum Meer Rownaq Ali, Manar Mahmoud Abed, Wadha Saad Albishi, Fathima Adnan Al Hajji, Najla Abdullah Sultan

**Affiliations:** 1 Department of Prosthodontics/Dental Lab Technology, College of Applied Medical Sciences, Riyadh Elm University, Riyadh, SAU

**Keywords:** saudi arabia, attitude, knowledge, dental practitioner, 3d printing

## Abstract

Background*: *This knowledge, attitude, and practices (KAP) survey will provide baseline data and identify gaps that may facilitate understanding and further action to plan, implement, and evaluate practice toward 3D-printing technology among dental practitioners in Saudi Arabia.

Aims and objectives: The present study aims to assess dental practitioners' self-reported knowledge, attitude, and practice of 3D printing in Saudi Arabia.

Methodology: A cross-sectional, closed-ended questionnaire of registered dental practitioners in Saudi Arabia was conducted. A sample size of 156 was considered for analysis. After obtaining approval from the Institutional Review Board, Riyadh Elm University, the research was conducted during the month of April 2022 amongst 154 registered dental practitioners. The research was distributed among dental health specialists either working in dental colleges, dental clinics, or both in government as well as private settings. Dentists who were not actively involved in 3D printing were excluded. SPSS software, version 25.0, (IBM Corp., Armonk, NY) was used to analyze the data.

Results and conclusion: Of all dentists included in the study, 98% were found to be aware that 3D printing in dentistry is used in Saudi Arabia and 2 % were not aware of its usage in Saudi Arabia. In total, 78.60% of the dentists felt that 3D-printed implant guides made the placement of implants the most accurate and least complicated procedure, and 21.40% of the dentists felt it was the least accurate and most complicated procedure.

## Introduction

After the first 3D (three-dimensional) printing technology was released in 1986, the manufacturing sector created a wide range of production techniques that have been used in a wide range of industries [1-4]. Hull created and developed a 3D printing method and obtained the stereolithography (SLA) patent in 1986 [4]. Fused deposition modeling (FDM) was the subject of a patent granted to Scott Crump in 1990 [4]. Since then, 3D printing has advanced significantly. 3D printing, which is based on digital computer-aided design (CAD) models, uses standardized materials to produce customized 3D objects using predetermined automatic procedures [5-7]. The traditional techniques of experimentation and instruction are likely to be dramatically altered by 3D printing due to the quick development of new materials, printing technology, and equipment. 3D printing is frequently employed in the medical profession in specialties like traumatology, cardiology, neurosurgery, plastic surgery, and craniomaxillofacial surgery [8]. Its applications in dentistry span a variety of disciplines, including prosthodontics, oral and maxillofacial surgery, and oral implantology [9].

This technique has numerous benefits and advantages over other techniques in process engineering [10]. Complete dentures and implant teeth are more accessible because of their quick fabrication, high level of precision, and ability to be personalized. Its use in dentistry can also help to streamline the difficult workflow involved in creating dental appliances and offer patients more individualized [11]. For instance, the restoration was often made by milling until 3D printing technology became widely used. Currently, 3D-printed restorations have demonstrated a number of benefits. Studies have revealed that 3D printed restorations have much greater marginal fit and accuracy. For instance, dental crowns are often made on the basis of conventional plaster models that have less accuracy when compared to digital scans and then preparation of the restoration [12]. 

The CAD data can be quickly read by the 3D printer. Additionally, it can quickly produce samples, complex-shaped goods, molds, and models in addition to single and small-batch parts [13]. Numerous benefits include high resource usage, significant cost gains, and the ability to produce specific-scale items as needed. It still has a number of drawbacks, though, including expensive processing and material costs and time-consuming post-processing. But 3D printing has been used in the medical field in a good way most of the time [14]. The development of Computer-Aided Design and Computer-Aided Manufacturing (CAD-CAM) and CBCT (Cone Beam Computer Tomography) technologies has led to a revolution in the use of this technique in the fields of medicine and dentistry [15-18]. Temporary crowns can be made more precisely using 3D printing than using the traditional technique [19]. It is therefore important that, as part of the future workforce, this knowledge, attitude, and practice (KAP) survey will provide baseline data and identify gaps that may facilitate understanding and further action to plan, implement, and evaluate practice toward 3D printing technology among dental practitioners in Saudi Arabia.

## Materials and methods

This research study protocol was submitted for ethical committee clearance/exemption from Riyadh Elm University (Registration number FRP/2021/422/682) and was approved with the approval number FRP/2021/422/682/659. The present study was conducted in Saudi Arabia among registered dental practitioners in Saudi Arabia. Non-probability A convenient sampling method was employed. Anonymity and informed consent of all the study participants were maintained. A cross-sectional, closed-ended questionnaire survey of registered dental practitioners in Saudi Arabia was conducted. A sample size of 154 was considered for analysis. After obtaining approval from the Institutional Review Board, Riyadh Elm University the study was started during the month of February 2022. The SurveyMonkey questionnaire for the present study was developed from the previous study [15], and the validity of the questionnaire was established by the pilot study. The sampling frame consisted of dentists registered as health practitioners in the Saudi Commission for Health Specialties either working in dental colleges, dental clinics or both in government, as well as private settings in Saudi Arabia, were the dental professionals. Dentists willing to participate voluntarily were included in this study, ensuring confidentiality. Using a link created programmatically, the online survey was delivered by the SurveyMonkey form. Dentists who are not actively involved in 3-D printing technology or who declined to participate after being contacted through the mail were excluded. Data were entered and analyzed using the Statistical Package for Social Sciences (SPSS), Statistics for Windows, version 25.0. (IBM Corp., Armonk, NY). For categorical data comparison, the Chi-square and Fisher's exact tests were used. A p-value of 0.05 at 95% CI (Continuous Integration) was considered statistically significant.

## Results

According to this study, 63.60 percent of dentists believe that 3D printing is employed in Saudi Arabia for dental purposes, while just 11 percent disagree. Seminars and presentations taught 15.60% of dentists about 3D printing, colleges taught 34.40%, guest lectures taught 8.4 percent, colleagues taught 20.80%, and clinical practice taught 20.80%. In total, 10.40% of dentists disagreed with the statement that 3D printing has recently become more common, while 66.20% agreed (Table 1).

**Table 1:** Characteristics of the study participants PhD: Doctor of Philosophy

Characteristics		n	%
Age (years)	18-24	38	24.7%
	25-34	82	53.2%
	35-44	29	18.8%
	55-64	5	3.2%
	Total	154	100.0%
Gender	Male	97	63.0%
	Female	57	37.0%
	Total	154	100.0%
Address	North of Saudi Arabia	26	16.9%
	South of Saudi Arabia	11	7.1%
	East of Saudi Arabia	32	20.8%
	West of Saudi Arabia	12	7.8%
	Central of Saudi Arabia	73	47.4%
	Total	154	100.0%
Educational qualification	Bachelor of dental surgery	115	74.7%
	Masters	30	19.5%
	PhD	9	5.8%
	Total	154	100.0%
Occupation	Faculty in Dental College	52	33.8%
	Private Practitioner	63	40.9%
	Both	22	14.3%
	Government. Job	17	11.0%
	Total	154	100.0%
Specialized maxillofacial or implantology	Yes	48	31.2%
	No	106	68.8%
	Total	154	100.0%

In response to questions on the biocompatibility and negative side effects of the 3D printed materials used in dentistry, 48.40% felt that they were safe, 11% felt they were unsafe, and 30.50% thought they might be. In terms of biocompatibility, quality, and functionality, studies on 3D-printed appliances and prostheses revealed that they are similar to currently used conventional techniques in the dental technology laboratory [20-21]. When asked why 3D printing was less common in Saudi Arabia than in other nations, dentists gave a variety of answers: 24.70% said there was a lack of knowledge about the technology, 27.90% said it was because the equipment was expensive, 11.70% said it was because complex techniques were required, and 35.70% said they had considered every possibility. When asked about the benefits of performing surgery using these models in cases of complex craniofacial fractures and surgeries, 26% of the dentists thought that surgery became more accurate and predictable, 20.80% thought that less time was spent on the operating table, 9.70% thought that patient safety was ensured, and 43.50% thought that all of the above reasons were true. According to the texts, these models provide intra-operative management and pre-operative treatment planning [22-23]. When asked about the use of 3D printed implant guides, 78.60% of dentists said it made the placement of implants the most accurate and least complicated operation, while 21.40% of dentists believed it made it the least accurate and most complicated process. In cases of calcified pulp canals, root canal therapy using 3D printed drill guides and templates may be helpful, according to 50.60 percent of dentists, whereas 14.90 percent disagreed. 34.40 percent said it could be put to use. Numerous cases of successful therapy have been carried out using this methodology [24-25]. When inquired how well they were aware that the Invisalign clear aligners used in orthodontics are 3D printed, 51.30% said yes, 21.40% said no, and 27% said maybe. When asked if they would be willing to explore the potential uses of this technique in dentistry further, 61.70 percent of dentists replied yes, 26.60 percent said maybe, and just 11.70 percent disagreed (Table 2).

**Table 2:** Questionnaire items and responses on 3D printing amongst the study participants (n=154).

Items		Responses		
		Yes	No	Maybe
Do you believe Saudi Arabia uses 3D printing for dental purposes?	n	98	17	39
	%	63.60%	11.00%	25.30%
Has its use in dentistry increased recently?	n	102	16	36
	%	66.20%	10.40%	23.40%
Do you believe that materials obtained through this technique will be biocompatible and free from negative patient side effects?	n	90	17	47
	%	58.40%	11.00%	30.50%
Do you believe that 3D printed drill templates and guides might be helpful when doing root canal treatments on pulp canals that have calcified?	n	78	23	53
	%	50.60%	14.90%	34.40%
Are you aware that the clear aligners used in Invisalign orthodontic treatment are 3D printed?	n	79	33	42
	%	51.30%	21.40%	27.30%
Would you like to discuss the uses of this technique in dentistry in more detail?	n	95	18	41
	%	61.70%	11.70%	26.60%

The questionnaire items did not reveal any significant association with the characteristics of the study participants (p>0.05) (Table 3). 

**Table 3:** Cross-tabulation between questionnaire items and characteristics of the study participants KSA: Kingdom of Saudi Arabia; BDS: Bachelor of dental surgery

Items		3D printing in KSA			3D Printing Popularity			3D printing biocompatibility			3D Invisalign			3D printing in dentistry		
		Yes	No	Maybe	Yes	No	Maybe	Yes	No	Maybe	Yes	No	Maybe	Yes	No	Maybe
		%	%	%	%	%	%	%	%	%	%	%	%	%	%	%
Age	18-24	19.4	17.6	41.0	24.5	31.3	22.2	24.4	23.5	25.5	26.6	21.2	23.8	24.2	11.1	31.7
	25-34	58.2	52.9	41.0	51.0	62.5	55.6	52.2	64.7	51.1	46.8	69.7	52.4	52.6	66.7	48.8
	>35	22.4	29.4	17.9	24.5	6.3	22.2	23.3	11.8	23.4	26.6	9.1	23.8	23.2	22.2	19.5
	p	.094			.583			.840			0.208			.539		
Gender	Male	59.2	64.7	71.8	62.7	68.8	61.1	63.3	64.7	61.7	59.5	57.6	73.8	60.0	61.1	70.7
	Female	40.8	35.3	28.2	37.3	31.3	38.9	36.7	35.3	38.3	40.5	42.4	26.2	40.0	38.9	29.3
	p	0.381			0.867			0.971			0.230			0.485		
Education	BDS	74.5	64.7	79.5	72.5	68.8	83.3	71.1	70.6	83.0	72.2	75.8	78.6	68.4	83.3	85.4
	≥Masters	25.5	35.3	20.5	27.5	31.3	16.7	28.9	29.4	17.0	27.8	24.2	21.4	31.6	16.7	14.6
	p	0.503			0.374			0.291			0.732			0.076		
Address in KSA	North	19.4	5.9	15.4	20.6	12.5	8.3	21.1	5.9	12.8	22.8	12.1	9.5	20.0	22.2	7.3
	South	9.2	0.0	5.1	6.9	18.8	2.8	6.7	17.6	4.3	3.8	15.2	7.1	9.5	0.0	4.9
	East	25.5	17.6	10.3	24.5	12.5	13.9	18.9	41.2	17.0	20.3	18.2	23.8	20.0	11.1	26.8
	West	6.1	11.8	10.3	5.9	6.3	13.9	5.6	0.0	14.9	5.1	12.1	9.5	7.4	11.1	7.3
	Central	39.8	64.7	59.0	42.2	50.0	61.1	47.8	35.3	51.1	48.1	42.4	50.0	43.2	55.6	53.7
	p	.189			.116			.053			.274			.417		
Occupation	Dental faculty	35.7	29.4	30.8	37.3	12.5	33.3	36.7	17.6	34.0	40.5	21.2	31.0	38.9	11.1	31.7
	Private Practitioner	43.9	35.3	35.9	41.2	43.8	38.9	41.1	41.2	40.4	41.8	42.4	38.1	41.1	38.9	41.5
	Both	14.3	23.5	10.3	11.8	31.3	13.9	13.3	23.5	12.8	11.4	21.2	14.3	11.6	33.3	12.2
	Government Job	6.1	11.8	23.1	9.8	12.5	13.9	8.9	17.6	12.8	6.3	15.2	16.7	8.4	16.7	14.6
	p	.141			.350			0.695			.262			.111		
Specialist max facial implants	Yes	36.7	17.6	23.1	32.4	50.0	19.4	36.7	35.3	19.1	32.9	30.3	28.6	27.4	44.4	34.1
	No	63.3	82.4	76.9	67.6	50.0	80.6	63.3	64.7	80.9	67.1	69.7	71.4	72.6	55.6	65.9
	p	0.132			0.081			0.102			0.880			0.319		

However, it was shown that the study participants' occupation (p=0.032) and speciality (p=0.002) were strongly associated with the reason that this approach is not frequently employed in Saudi Arabia (Table 4).

**Table 4:** Cross-tabulation between questionnaire items and characteristics of the study participants KSA: Kingdom of Saudi Arabia; BDS: Bachelor of dental surgery

Variables		Source of information on 3D					Reason for 3D printing not widely used in Saudi Arabia			
		Colleges	Clinical practice	Colleagues	Guest lectures	Seminars	Lack of awareness	Cost	Complex	All
		%	%	%	%	%	%	%	%	%
Age	18-24	28.3%	15.6%	31.3%	23.1%	20.8%	26.3%	16.3%	33.3%	27.3%
	25-34	52.8%	62.5%	50.0%	46.2%	50.0%	42.1%	69.8%	44.4%	50.9%
	>35	18.9%	21.9%	18.8%	30.8%	29.2%	31.6%	14.0%	22.2%	21.8%
	p	0.838					0.223			
Gender	Male	67.9%	59.4%	59.4%	84.6%	50.0%	55.3%	60.5%	72.2%	67.3%
	Female	32.1%	40.6%	40.6%	15.4%	50.0%	44.7%	39.5%	27.8%	32.7%
	p	0.262					0.536			
Education	BDS	73.6%	75.0%	75.0%	69.2%	79.2%	63.2%	81.4%	61.1%	81.8%
	≥Masters	26.4%	25.0%	25.0%	30.8%	20.8%	36.8%	18.6%	38.9%	18.2%
	p	0.974					0.074			
Address	North	22.6%	18.8%	15.6%	0.0%	12.5%	23.7%	14.0%	22.2%	12.7%
	South	7.5%	9.4%	9.4%	7.7%	0.0%	10.5%	4.7%	16.7%	3.6%
	East	20.8%	21.9%	25.0%	23.1%	12.5%	18.4%	27.9%	22.2%	16.4%
	West	5.7%	12.5%	6.3%	7.7%	8.3%	5.3%	7.0%	0.0%	12.7%
	Central	43.4%	37.5%	43.8%	61.5%	66.7%	42.1%	46.5%	38.9%	54.5%
	p	.772					.399			
Occupation	Dental faculty	45.3%	18.8%	28.1%	46.2%	29.2%	44.7%	23.3%	22.2%	38.2%
	Private Practitioner	35.8%	56.3%	31.3%	38.5%	45.8%	39.5%	58.1%	38.9%	29.1%
	Both	11.3%	12.5%	18.8%	7.7%	20.8%	13.2%	14.0%	22.2%	12.7%
	Govt. Job	7.5%	12.5%	21.9%	7.7%	4.2%	2.6%	4.7%	16.7%	20.0%
	p	.215					.032			
Specialized maxillofacial implants	Yes	37.7%	28.1%	37.5%	15.4%	20.8%	50.0%	23.3%	50.0%	18.2%
	No	62.3%	71.9%	62.5%	84.6%	79.2%	50.0%	76.7%	50.0%	81.8%
	p	0.342					.002^*^			

## Discussion

Different types of malocclusion have a negative impact on dental cleanliness and periodontal health as well as dental aesthetics and a beautiful smile. Patients seek orthodontic treatment for a variety of reasons, such as health/dental issues, to improve their look, and to increase their confidence and self-esteem [26]. Orthodontic therapy using aligners is becoming more and more popular among patients and doctors because it is not only efficient but also doesn't interfere with their daily lives. Compared to traditional orthodontics, this therapy uses less force to move the teeth. These devices seem unattractive and cause irritation on the lips [27-28].

This particular type of brace is becoming more and more popular because of the limitations of conventional therapy and patients' increased desire for "invisible" treatments [29-30]. The earliest clear aligners were created as a technique only for minor tooth position anomalies or for use in the last stages of orthodontic therapy [31]. Eventually, advancements in the field of clear aligners made it possible to cure moderate to severe malocclusions with them. Since there hasn't been much research done to introduce new materials specifically for this purpose, clear aligner orthodontic techniques have evolved using materials that are currently on the market.

For dental applications such as dental implants, craniofacial, maxillofacial, orthognathic, and periodontal treatments, endodontic non-surgical and surgical treatments, and the development of implant copings and frameworks and dental restorations, 3D-printing techniques primarily include selective laser printing and fused deposit modeling.

The creation of an anatomical "study model" can be considered one of the early uses of 3D printing in dental surgery [32]. Due to the widespread availability of CBCT in dental offices, which has revolutionized diagnosis and treatment in endodontics and implant dentistry, this crucial technology has been made even more accessible in recent years. Before surgery, volumetric data can be sent to a 3D printer in order to create accurate copies of the patient's jaws [33]. This is made possible by the availability of CT or CBCT, which both give similar data and are more common in a hospital context [34]. This enables a surgical strategy to be planned out or practiced before surgery, especially for complex, unique, or unknown anatomy.

It is feasible to create a precise virtual model of the prepared tooth, implant position, and dental arch using intraoral optical scanners or laboratory scanners. Treatment can be planned and restorations can be created using computer-aided design tools in fixed and removable prosthodontics. Implant abutments, bridge constructions, and crown or bridge copings can all be milled or printed using the scan data and design. The construction of dentures has become a more amiable process for patients since the advent of intraoral scanning and 3D printing [35]. According to published case reports, patients with restricted mouth opening or lip contractures can now successfully have detachable partial dentures made [36]. Fixed and removable dentures created using 3D printing are clinically acceptable and have similar physical characteristics to dentures created using more traditional methods [37]. According to studies, metal implant prostheses can be successfully printed utilizing 3D technology and selective laser melting or electron beam melting [38]. By using this cutting-edge technology, dental technicians can do less laborious work while producing a framework that is more exact than a traditional framework.

Regarding the field of periodontics, laser sintering and direct beam melting have emerged as the cutting edge in the production of specialized porosity implants such as specialized titanium mesh and reconstruction. While milling/machining may also be used to refine the printed shape, such as the implant platform, 3D printing has the capacity to construct complicated geometries, such as a bone-like morphology, that may not be possible to produce using milling alone [39]. Though finally placing a dental implant using a screw-like form seems to be a well-proven method, there is also the possibility of constructing implants that have complex geometry. Based on the needs of the defect area, many 3D printing processes find application in tissue regeneration [40]. The template for making 3D objects is a CT scan of the patient's defect. A printed wax mold is created using the CT picture to create a scaffold that can be utilized to enhance the immigration of periodontal ligament cells, which are in charge of fusing dental cementum and tooth roots [41]. In a nutshell, the primary goal of the use of 3D printing technology in oral implantology and periodontics is to transform these fields from analog, experience-based modes to digital, precise modes. They can considerably reduce the technical complexity and risk, streamline the medical treatment procedure, and boost dentists' effectiveness.

Talking about the limitations of our investigation, we think our study's relatively small sample size is the only one that can be attributed to it. To prove that 3D printing is a useful tool for dental procedures and dental professionals alike, more research is, in our opinion, required.

## Conclusions

A growing number of material types and a reliance on digital data are made possible by 3D printing. The virtue of using CAD software based on this procedure and imaging is that it has a high substance utilization rate, which allows for the construction of complex structures. Data on the patient is digitally saved and only printed when necessary to reduce the demand for physical space. However, this method has some limitations, including the exorbitant expense of the equipment, the difficulty to be employed in crisis situations, and the impossibility to create very large items. Even while dentists in Saudi Arabia are aware of the use of these advanced techniques in dentistry, there is still a dearth of knowledge about the technology.
